# Clinical characteristics of patients diagnosed with bilateral sudden sensorineural hearing loss

**DOI:** 10.3389/fneur.2024.1378017

**Published:** 2024-06-24

**Authors:** Jun He, Li Jin, Jacqueline Yao, Ahmad Mahmoudi, Zhen Pan, Jinfeng Fu, Qiulin Yuan, Wei Liu

**Affiliations:** ^1^Department of Otolaryngology-Head and Neck Surgery, The Second Xiangya Hospital, Central South University, Changsha, China; ^2^Department of Otolaryngology-Head and Neck Surgery, Stanford University School of Medicine, Stanford, CA, United States; ^3^Department of Otolaryngology-Head and Neck Surgery, Changde Hospital, Xiangya School of Medicine, Central South University (The First People’s Hospital of Changde City), Changde, China

**Keywords:** bilateral sudden sensorineural hearing loss (BSSNHL), sudden hearing loss, etiology, risk factor, prognosis

## Abstract

This study investigated the etiology, clinical features, and prognosis of patients diagnosed with bilateral sudden sensorineural hearing loss (BSSNHL). The clinical data of 100 patients with bilateral sudden hearing loss as a chief complaint treated at Xiangya Second Hospital of Central South University between January 2010 and August 2022, including clinical characteristics, audiometric data, and prognosis, were retrospectively analyzed. These 100 cases accounted for 8.09% (100/1235) of all patients admitted for sudden sensorineural hearing loss (SSNHL) during the same period. Of these, 71 were simultaneous cases and 29 were sequential cases of BSSNHL. Among the 200 ears analyzed in this study, 13, 36, 57, and 94 had mild, moderate, severe, and profound sensorineural hearing loss, respectively. The overall effective rate after comprehensive treatment was 32%, with significant differences in efficacy and prognosis among different degrees of hearing loss (*p* < 0.05). Comorbidities of hypertension (24 cases), diabetes (14 cases), and coronary heart disease (9 cases) significantly impacted therapeutic efficacy and prognosis in patients with BSSNHL (*p* < 0.05). Compared to unilateral SSNHL, BSSNHL exhibits distinctive characteristics.

## 1 Introduction

Sudden sensorineural hearing loss (SSNHL) is an acute, unexplained hearing loss occurring within 72 h. Criteria for a SSNHL diagnosis varies; the latest Chinese guidelines define SSNHL as ≥20 dB of hearing loss in two consecutive frequencies (1) and the American Academy of Otolaryngology-Head and Neck Surgery defines SSNHL as hearing loss ≥30 dB at three consecutive frequencies (2). Clinically, SSNHL is categorized into unilateral SSNHL (USSNHL) and bilateral SSNHL (BSSNHL). The incidence of BSSNHL is considerably lower than that of USSNHL (3, 4). Based on the time interval between the onset of hearing loss in both ears, BSSNHL is further divided into simultaneous BSSNHL (Si-BSSNHL, onset in both ears within ≤3 days) and sequential BSSNHL (Se-BSSNHL, onset in both ears with an interval of >3 days) (4). Existing evidence suggests that BSSNHL differs from USSNHL in etiology, treatment, and prognosis (5). USSNHL is often idiopathic and has higher recovery rates (6). In contrast, BSSNHL is frequently associated with underlying systemic diseases, leading to more profound hearing loss and less favorable treatment outcomes (5–7). Given the heterogeneity of BSSNHL etiologies and its low incidence, BSSNHL remains relatively poorly understood (2). This study aims to retrospectively analyze the clinical data of patients diagnosed with BSSNHL and summarize the clinical characteristics, etiologies, treatment outcomes, and factors influencing prognosis.

## 2 Materials and methods

### 2.1 Retrospective study design and patient selection

Clinical data were gathered from 100 patients diagnosed with BSSNHL who were treated at the Department of Otolaryngology, Head, and Neck Surgery, the Second Xiangya Hospital of Central South University, between January 2010 and August 2022. The inclusion criteria were as follows: (1) confirmed SSNHL with audiometry; (2) sudden onset of ≥20 dB HL within 72 h, affecting at least two consecutive frequencies (1); (3) simultaneous or sequential involvement of both ears. Patients previously diagnosed with Meniere’s Disease were excluded from the study. A retrospective analysis encompassed patients’ general information, physical exam findings, audiometric examinations, laboratory tests, imaging evaluations, and details regarding treatment and prognosis. We excluded patients who were lost to follow-up.

Pre- and post-treatment, all patients underwent standard audiometric assessments, including pure-tone audiometry, impedance audiometry, auditory brainstem response, and otoacoustic emissions. High-resolution computed tomography of the temporal bone was conducted to rule out middle and inner ear diseases. A brain magnetic resonance imaging (MRI) examination was performed to rule out intracranial diseases. Serological tests included C-reactive protein, neutrophil count, lymphocyte count, monocyte count, platelet count, and fibrinogen level. Antibody tests included immunoglobulin G, immunoglobulin M, immunoglobulin A, complement 3 and complement 4, antinuclear antibody, antineutrophil cytoplasmic antibodies (ANCA), and rheumatoid factor. Biochemical tests included total bilirubin, indirect bilirubin, blood glucose, triglyceride, total cholesterol, high-density lipoprotein, low-density lipoprotein. Patients were also administered tests for human immunodeficiency virus (HIV), syphilis, hepatitis B, hepatitis C, and herpes virus.

This study was conducted by the principles of the Declaration of Helsinki and approved by the Ethics Committee of Second Xiangya Hospital.

### 2.2 Audiologic evaluation

Based on pure-tone audiometry results, the pure-tone average of air-conduction hearing thresholds was calculated at four frequencies (500 Hz, 1,000 Hz, 2000 Hz, and 4,000 Hz). Subsequently, hearing loss was classified according to the WHO proposed hearing-impairment grading system (2008) (8) as follows: mild: 26–40 dB HL; moderate: 41–60 dB HL; severe: 61–80 dB HL; and profound: ≥81 dB HL.

### 2.3 Treatment methods

Adhering to the 2015 Chinese Medical Association guidelines for SSNHL (1), the patients’ treatment protocol involved intravenous administration of dexamethasone at 10 mg/day for adults, with pediatric dosage calculated based on their body weight. This treatment was administered continuously for 3 days; if deemed effective, an additional 2-day course was administered before discontinuation. For patients with contraindications to systemic corticosteroid use or insufficient systemic corticosteroid efficacy, intratympanic dexamethasone perfusion at 5 mg/session was administered once every other day for a total of four to five sessions. Additionally, depending on the patient’s condition, treatment was complemented with neurotrophic agents (such as methylcobalamin), drugs enhancing inner ear microcirculation (such as *Ginkgo biloba* leaf extract), antioxidants, fibrinolysis inhibitors, or hyperbaric oxygen therapy. For patients with identified SSNHL etiologies, specific treatments were integrated alongside the aforementioned therapies.

Treatment efficacy was evaluated by pure-tone audiometry results 3 months after standard treatment (1). When evaluating the efficacy of Si-BSSNHL, it is considered effective if one of the two ears shows improvement; if both ears are ineffective, it is considered ineffective. When evaluating the efficacy of Se-BSSHL, the effectiveness is based on the second ear. Further sub-categories include (1) curative: average hearing thresholds normalizing or reverting to pre-illness levels; (2) significantly effective: average hearing improvement of >30 dB at the specified frequencies; (3) effective: average hearing improvement of 15–30 dB at the specified frequencies; and (4) ineffective: average hearing improvement of <15 dB at the specified frequencies.

### 2.4 Statistical analysis

Statistical analysis was conducted using SPSS 26.0 software (IBM Corp., Armonk, NY, United States). Kruskal–Wallis test was applied for continuous data analysis, while categorical data were analyzed using chi-square, or Fisher’s exact probability tests, depending on the circumstances. The level of significance was set at values of *p* < 0.05.

## 3 Results

### 3.1 General information and clinical characteristics

Among the 100 patients diagnosed with BSSNHL, 52 patients were male and 48 were female. Patients’ ages ranged from 2 to 86 years, with an average age of 46.28 ± 19.77 years. Eighty-nine patients were adults, and 11 patients were children. Triggers, such as respiratory infections, exposure to cold, and physical exertion, were reported by 20% of the patients before the BSSNHL onset. Of the 100 patients, 71 and 29 patients had Si-BSSNHL and Se-BSSNHL, respectively. In the Si-BSSNHL group, 44 patients reported tinnitus, 5 reported ear fullness, 31 reported dizziness/vertigo, and 17 reported nausea/vomiting. Sixteen Si-BSSNHL patients had hypertension (22.5%), 12 had diabetes (16.9%), and 7 had coronary heart disease (9.9%). In the Se-BSSNHL group, 21 patients reported tinnitus, 2 reported ear fullness, 10 reported dizziness/vertigo, and 6 reported nausea/vomiting. Eight Se-BSSNHL patients had hypertension (27.6%), two had diabetes (6.9%), and two had coronary heart disease (6.9%). Some patients have also reported ear pain, headache, unsteady gait, speech impairment, photophobia, rhinorrhea, nasal congestion, and depression.

When comparing clinical characteristics between the two groups of BSSNHL patients, no statistically significant differences were observed in sex, age, presence of tinnitus, ear fullness, dizziness/vertigo, nausea/vomiting symptoms, concurrent hypertension, diabetes, and coronary heart disease (*p* > 0.05) (Table 1).

**Table 1:** Clinical characteristics of patients diagnosed with BSSNHL.

Variables	Si-BSSNHL (*n* = 71)	Se-BSSNHL (*n* = 29)	*p*-value
**Sex**			
Male	38 (53.5%)	14 (48.3%)	0.634^C2^
Female	33 (46.5%)	15 (51.7%)	
**Age**	44.85 ± 19.37	49.79 ± 20.64	0.372^H^
**Triggers**	13 (65%)	7 (35%)	0.634^C2^
**Accompanying symptoms**			
Tinnitus	44 (62.0%)	21 (72.4%)	0.321^C2^
Ear fullness	5 (7.0%)	2 (6.9%)	>0.9^F^
Dizziness/vertigo	31 (43.7%)	10 (34.5%)	0.397^C2^
Nausea/vomiting	17 (23.9%)	6 (20.7%)	0.726^C2^
**Comorbidities**			
Hypertension	16 (22.5%)	8 (27.6%)	0.592^C2^
Diabetes mellitus	12 (16.9%)	2 (6.9%)	0.340^F^
Coronary heart disease	7 (9.9%)	2 (6.9%)	>0.9^F^

^C2^Chi-square test; ^H^Kruskal–Wallis test; ^F^Fisher’s exact tests. BSSNHL, bilateral sudden sensorineural hearing loss; Si-BSSNHL, simultaneous BSSNHL; Se-BSSNHL, sequential BSSNHL.

### 3.2 Etiological distribution

Possible SSNHL etiologies were identified in 38 of all patients evaluated in this study (38%) (Table 2). Vascular, autoimmune, and infectious diseases were identified in 15, 6, and 5 patients, respectively. Neoplasms, large vestibular aqueduct syndrome (LVAS), and uremia was diagnosed in 2, 7, and 3 patients, respectively. The etiologies for the remaining 62 patients were classified as idiopathic. Five of the 11 pediatric patients had LVAS, which was preceded by falls and head trauma in all 5 patients.

**Table 2:** Etiologies of patients with diagnosed with BSSNHL.

Pathogenesis	Si-BSSNHL	Se-BSSNHL	Total
**Vascular disease**	8	7	15
Stroke	7	4	11
Sigmoid sinus thrombosis	1	—	1
Craniofacial vascular malformation	—	1	1
Bilateral lower limb venous thrombosis	—	2	2
**Autoimmune disease**	3	3	6
Antiphospholipid syndrome	1	—	1
Relapsing polychondritis	1	1	2
Sjögren’s syndrome	—	1	1
Vogt–Koyanagi–Harada disease	1	—	1
Cogan syndrome	—	1	1
**Infectious disease**	4	1	5
HIV	2	—	2
HIV coexisting with syphilis	—	1	1
Bacterial meningitis	2	—	2
**Neoplastic disease**	2	—	2
Neurofibromatosis type 2	1	—	1
Acute myeloblastic leukemia with maturation (AML-M2)	1	—	1
**Large vestibular aqueduct syndrome**	7	—	7
**Uremia**	3	—	3
**Idiopathic**	44	18	62
**Total**	71	29	100

BSSNHL, bilateral sudden sensorineural hearing loss; Si-BSSNHL, simultaneous BSSNHL; Se-BSSNHL, sequential BSSNHL; HIV, human immunodeficiency virus.

### 3.3 Audiometric examination results

**Figure 1:**
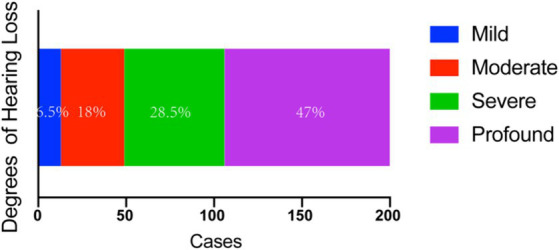
Audiometric examination results with BSSNHL. The main hearing loss type is severe (28.5%) and profound (47%) HL. BSSNHL, bilateral sudden sensorineural hearing loss.

In the total cohort of 100 patients and 200 ears tested, mild sensorineural hearing loss was observed in 13 ears (6.5%), moderate sensorineural hearing loss in 36 ears (18.0%), severe sensorineural hearing loss in 57 ears (28.5%); and profound sensorineural hearing loss in 94 ears (47.0%) (Figure 1).

### 3.4 Treatment outcomes

**Figure 2:**
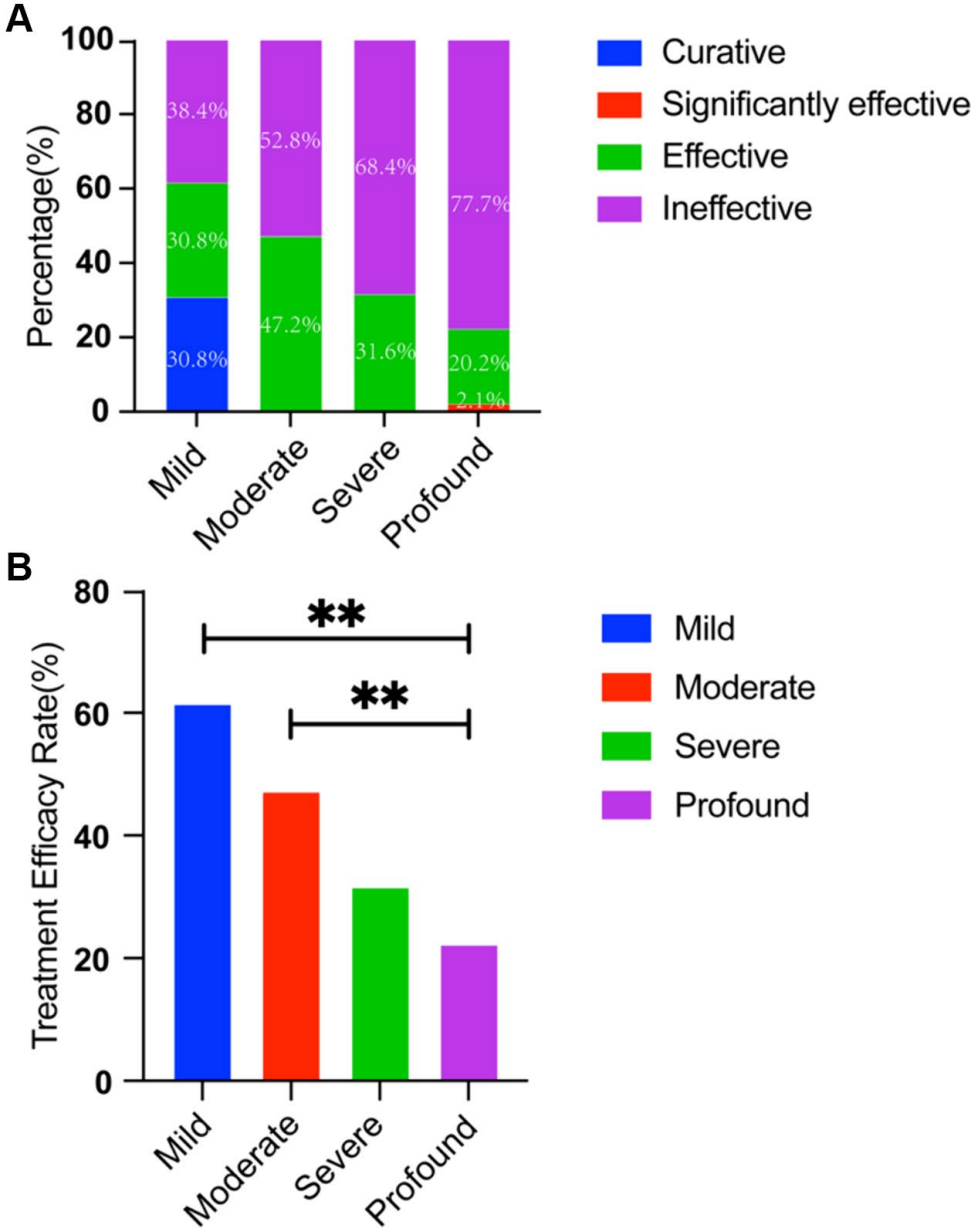
**(A)** Comparison of efficacy in patients with BSSNHL at different degrees of hearing loss. Treatment outcomes deteriorates as the severity of hearing loss increases. **(B)** The treatment efficacy for profound sensorineural hearing loss was significantly lower than that of mild sensorineural hearing loss (*p* < 0.01) and moderate sensorineural hearing loss (*p* < 0.01). ^**^p < 001. BSSNHL, bilateral sudden sensorineural hearing loss.

Treatment outcomes of etiological therapy, steroid administration (systemic or intratympanic dexamethasone), and interventions to enhance microcirculation and neurotrophic support, were as follows: of the 200 ears, treatment was curative for 4 ears, significantly effective for 2 ears, effective for 58 ears, and ineffective for 136 ears, resulting in an overall efficacy rate of 32%. Significant differences in treatment efficacy were observed among patients with different degrees of hearing loss (*p* = 0.004). The treatment effect deteriorates as the severity of hearing loss increases (Figure 2A). The treatment efficacy in patients with mild, moderate, severe, and profound hearing loss was 61.6, 47.2, 31.6, and 22.3%, respectively. Patients with mild (*p* < 0.05) and moderate (*p* < 0.05) hearing loss demonstrated higher treatment efficacy than that of patients with profound hearing loss (Figure 2B).

**Figure 3:**
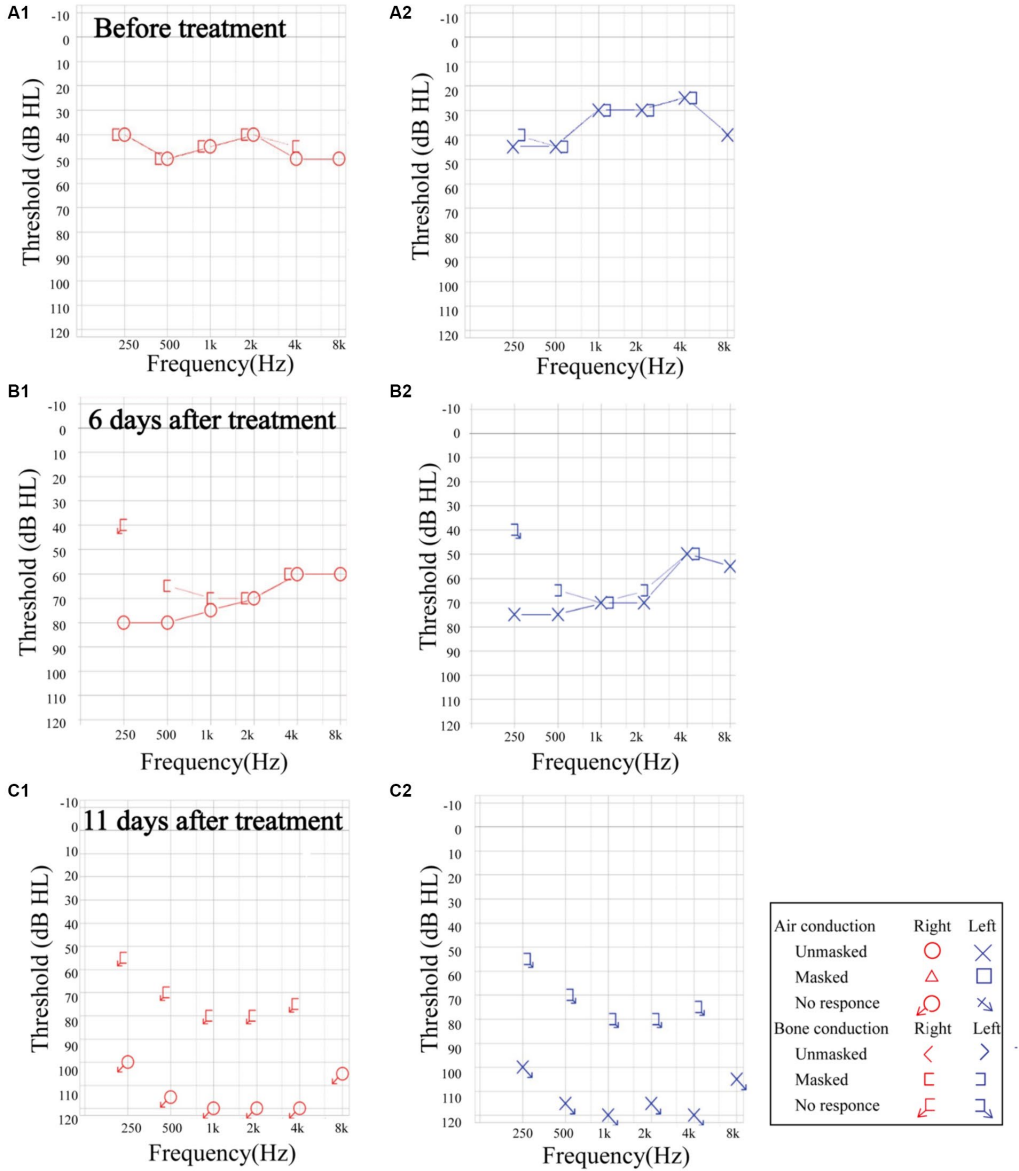
The progression of sensorineural hearing loss in a patient with HIV. **(A1,A2)** Before treatment, the pure tone audiometry threshold was 45.83 dB HL in the right ear. and 35.83 dB HL in the left ear. **(B1,B2)** At 6 days post-treatment, the unmasked hearing threshold increased across all frequencies. The average auditory threshold was 70.83 dB HL in the right ear, and 65.83 dB HL in the left ear. **(C1,C2)** At 11 days after treatment, the hearing threshold presented profound sensorineural hearing loss. The auditory threshold could not be tested bilaterally. HIV: human immunodeficiency virus.

The overall treatment efficacy rate in male patients was significantly higher than that in female patients (*p* < 0.05). Additionally, comorbidities of hypertension, diabetes, and coronary heart disease significantly decreased BSSNHL treatment efficacy (*p* < 0.05). Tinnitus, ear fullness, dizziness/vertigo, nausea/vomiting did not significantly affect treatment efficacy (*p* > 0.05) (Table 3). Moreover, we observed that some patients with BSSNHL were more likely to have progressive hearing loss, even during treatment. In one patient affected by HIV, the hearing threshold showed progressive decline (Figure 3).

**Table 3:** Analysis of clinical characteristics and treatment efficacy in patients diagnosed with BSSNHL.

Variables	Cases (*n* = 100)	Efficacy	*p*-value
**Sex**			
Male	52	24 (46.1%)	0.004^C2^
Female	48	9 (18.9%)	
**Classification**			
Si-BSSNHL	71	23 (32.4%)	0.84^C2^
Se-BSSNHL	29	10 (34.5%)	
**Tinnitus**	65	18 (27.7%)	0.124^C2^
**Ear fullness**	7	3 (42.9%)	0.681^C2^
**Dizziness/vertigo**	41	15 (36.6%)	0.525^C2^
**Nausea/vomiting**	23	7 (30.4%)	0.766^C2^
**Hypertension**	24	4 (16.7%)	0.032^C2^
**Diabetes mellitus**	14	2 (14.3%)	0.044^F^
**Coronary heart disease**	9	1 (11.1%)	0.026^F^

^C2^Chi-square test; ^F^Fisher’s exact tests. BSSNHL, bilateral sudden sensorineural hearing loss; Si-BSSNHL, simultaneous BSSNHL; Se-BSSNHL, sequential BSSNHL.

## 4 Discussion

BSSNHL is a rare otologic condition that can occur simultaneously or sequentially, presenting in only 0.57–14.5% of all patients with SSNHL (4–6, 9). In this study, the 100 patients diagnosed with BSSNHL without prior diagnosis of Meniere’s Disease constituted 8.09% (100/1235) of all patients admitted for SSNHL during the selected period. Clinical data from in-patients with BSSNHL was analyzed for the condition’s etiology and clinical characteristics, thereby offering insights into the diagnosis and treatment of BSSNHL.

Underlying etiologies were identified in 38/100 patients; of these, 7 patients (18.4%) were diagnosed with LVAS, the only otologic etiology in this patient group. Thirty-one patients were diagnosed with systemic conditions, including vascular diseases, autoimmune diseases, infectious diseases, neoplastic diseases, and uremia. Co-morbidities were not present in this study’s pediatric group.

Vascular diseases were the most common possible etiologies of BSSNHL in this study and were more prevalent in older patients with BSSNHL. The inner ear is supplied by blood from the labyrinthine artery, which branches into the cochlea-vestibular artery, cochlear artery, and the anterior vestibular artery (10). Existing literature indicates that cases of SSNHL with concurrent cerebral infarction are predominately caused by infarction in the anterior inferior cerebellar artery—from which the labyrinthine artery most commonly arises (11) with only a minority associated with infarctions in the posterior inferior cerebellar artery or vertebral-basilar artery (9). Evidently, thrombosis, vasospasm, bleeding, and other vascular diseases can disturb otovestibular microcirculation, causing auditory and vestibular functional impairment. Brain MRI and magnetic resonance angiography examinations often reveal corresponding ischemic lesions in the affected areas (9, 12). However, in the early stages of the disease, MRI examinations may indicate negative results and multiple evaluations may be necessary to identify the cause. Therefore, some patients with SSNHL and concurrent ischemia present with BSSNHL as the only symptom, whereas others experience symptoms more consistent with cerebral infarctions such as dizziness, nystagmus, ataxia, falls, and speech challenges. Clinical manifestations and prognosis are related to the location and severity of vascular lesions, with severe cases posing a potential threat to life (13). Therefore, in patients with BSSNHL, accompanying focal neurological symptoms or signs such as ataxia and speech challenges warrant a high level of vigilance for cerebral infarction (14).

Autoimmune diseases are a major cause of BSSNHL, with reported cases of bilateral sensorineural hearing loss associated with systemic lupus erythematosus, granulomatosis with polyangiitis, relapsing polychondritis, antiphospholipid syndrome, Behçet’s disease, and Sweet syndrome, among others (9, 15–18). In this study, the occurrence of BSSNHL in five patients may be attributed to autoimmune diseases, including one case of antiphospholipid syndrome, two cases of relapsing polychondritis, one case of Sjögren’s syndrome, one case of Vogt–Koyanagi–Harada disease, and one case of Cogan syndrome. A prospective cohort study found that the risk for SSNHL is significantly higher in patients with autoimmune diseases (i.e., multiple sclerosis, Behçet’s disease, antiphospholipid syndrome), which supports this study’s findings (19). The pathophysiology mechanism may involve vascular endothelial inflammation in the cochlea, leading to impaired blood supply (20).

LVAS is the most common cause of sensorineural hearing loss in children (21). Five (45%) pediatric patients in this study underwent high-resolution temporal bone computed tomography scans and were diagnosed with LVAS. Recurrence is possible, often with precipitating factors leading to inner ear pressure imbalance and disturbances in the internal environment before onset (21). Given the prevalence of LVAS, for pediatric patients presenting with BSSNHL, a detailed medical history examination and a high-resolution temporal bone CT examination are necessary to identify inner ear abnormalities.

The suspected BSSNHL etiology in two patients in this study is meningitis, a finding previously reported (22). Other identified infectious causes in the literature include COVID-19 (23) and Lyme disease (24). Three patients were diagnosed with HIV, a risk factor not previously extensively investigated as a risk factor for BSSNHL. Notably, the patient with an HIV presented with increased hearing loss after treatment. Clinicians should consider SSNHL as an additional potential complication in HIV patients.

Vestibular schwannomas, which are present in most patients with neurofibromatosis type 2, are highly prevalent in patients with SSNHL (1.12–4.0%) (25–27) compared to the general population. One patient (1%) in this study has been diagnosed with neurofibromatosis type 2, which is consistent with previous findings. To our knowledge, acute myeloblastic leukemia with maturation (AML-M2) has not been associated with SSNHL in previous studies. Given that one patient in our study is diagnosed with AML-M2 alongside BSSNHL, future research should investigate the role of non-vestibular neoplasms, such as AML-M2, in SSNHL pathophysiological and progression.

BSSNHL causes more significant hearing impairment and poorer treatment outcomes and prognosis compared to USSNHL, consistent with the findings of the present study (4, 5). The overall treatment efficacy in this study was 32%, which aligns with previous studies reporting a treatment efficacy for BSSNHL of 12.5–37.5% (7, 22, 28). Though consistent with previous studies, the relatively low efficacy and poorer prognosis in this study may also be associated with a higher proportion of patients with severe or profound sensorineural hearing loss. Among the 100 patients (200 ears), 75.5% (151/200) exhibited severe or profound sensorineural hearing loss, while only 24.5% (49/200) had mild or moderate sensorineural hearing loss. This indicates that patients with BSSNHL are more likely to experience a more severe degree of hearing loss. The study findings revealed significant differences in treatment efficacy and prognosis among different degrees of hearing loss (*p* = 0.004). The treatment efficacy for profound sensorineural hearing loss was lower than that of mild sensorineural hearing loss (*p* < 0.01) and moderate sensorineural hearing loss (*p* < 0.01), suggesting that the degree of hearing loss is a crucial factor influencing the efficacy and prognosis of BSSNHL. Most patients with effective treatment had idiopathic etiology (70%). Moreover, hearing loss usually presents with full-frequency descent, and patients with BSSNHL may have progressive hearing loss. It emphasizes the importance of paying attention to unexpected outcomes and poor prognosis during treatment to avoid further sequelae.

The most common accompanying symptom in BSSNHL patients is tinnitus, present in 65/100 of the BSSNHL patients in this study. The effect of tinnitus on BSSNHL prognosis varies in previous studies (28). In our study, tinnitus showed no statistically significant relevance to efficacy evaluation.

In this study, patients with BSSNHL and comorbidities such as hypertension, diabetes, and coronary heart disease accounted for 24, 14, and 9% of all participants, respectively. Furthermore, patients with these comorbidities had a poorer prognosis (*p* < 0.05). Aimoni et al. (29) indicated that cardiovascular diseases, diabetes, and metabolic disorders were risk factors for the onset of SSNHL and demonstrated an unfavorable impact on the prognosis, aligning with our study’s findings. It is important to note that other patient characteristics variables (i.e., age, gender) can confound the association between these chronic conditions and BSSNHL. Nonetheless, clinicians should be aware of hypertension, diabetes, and coronary heart disease as risk factors for poor treatment outcomes in BSSNHL patients.

## 5 Conclusion

In summary, BSSNHL exhibits distinctive clinical characteristics compared to USSNHL. BSSNHL usually manifests with severe sensorineural hearing loss which may progressively worsen. Vascular, autoimmune, infectious, neoplastic, and uremic were considered as potential pathogenesis in our study. BSSNHL is closely associated with underlying chronic conditions such as diabetes, hypertension, and coronary heart disease, all of which predict poor prognosis. Clinical diagnosis and treatment should involve a detailed medical history and early comprehensive systemic examinations to achieve a precise diagnosis and timely targeted treatment, preventing misdiagnosis or delayed treatment. Based on our findings, accounting for patient sex and other comorbidities can guide us toward the proper diagnostic tests to unravel the underlying cause of BSSNHL. Although early examination, diagnosis, and treatment are crucial, long-term and close follow-up visits are necessary for patients with an unclear etiology.

## Data availability statement

The raw data supporting the conclusions of this article will be made available by the authors, without undue reservation.

## Ethics statement

This study was conducted in accordance with the Declaration of Helsinki and approved by the Institutional Review Board of the local ethics committee at the Second Xiangya Hospital of Central South University (protocol code: LYF20230181, November 2023). Written informed consent for participation in this study was provided by the participants or their legal guardians/next of kin.

## Author contributions

JH: Investigation, Writing – original draft. LJ: Investigation, Writing – review & editing. JY: Formal Analysis, Writing – review & editing. AM: Formal Analysis, Writing – review & editing. ZP: Data curation, Writing – review & editing, Investigation. JF: Data curation, Writing – review & editing, Investigation. QY: Data curation, Writing – review & editing, Investigation. WL: Writing – review & editing, Supervision, Methodology, Funding acquisition.
